# Improvised Long Test Lengths via Stitching Scale Bar Method: Interim Testing of Laser Trackers

**DOI:** 10.6028/jres.125.016

**Published:** 2020-05-28

**Authors:** Vincent D. Lee, Daniel Sawyer, Bala Muralikrishnan

**Affiliations:** 1 National Institute of Standards and Technology, Gaithersburg, MD 20899, USA

**Keywords:** interim testing, laser scanners, laser tracker systems, scale bar

## Abstract

Performance verifications of laser tracker systems (LTSs) often rely on calibrated length artifacts that are 2.3 m in length or more, as specified in International Standards Organization (ISO) and American Society of Mechanical Engineers (ASME) standards. The 2.3 m length is chosen as the minimum length that will sufficiently expose inaccuracy in LTSs. Embodiment of these artifacts often comes in the form of scale bars, fixed monuments, or a laser rail. In National Institute of Standards and Technology (NIST) Internal Report (IR) 8016, which was published in 2014 and discusses interim testing of LTSs, it was shown that a scale bar with three nests spaced 1.15 m apart was sufficient for exposing errors in LTSs. In that case, the LTS was placed symmetrically with respect to the scale bar so that both a 2.3 m symmetrical length and a 1.15 m asymmetrical length were presented to the LTS. This paper will evaluate whether a scale bar that is only 1.15 m in length can sufficiently expose errors within the LTS when it is stitched together to create a 2.3 m long test length.

## 1 Introduction

Testing dimensional measurement systems against calibrated artifacts is one of the few ways that userscan obtain confidence in their measurement systems. There are a variety of methods to accomplish this, such as: testing in accordance to documentary standards such as those published by the American Society of Mechanical Engineers (ASME), ASTM International (formerly the American Society for Testing and Materials), or International Standards Organization (ISO), round robin testing, gauge reproducibility and repeatability, using a “golden part,” and interim testing using engineering procedures. In the case of laser tracker systems (LTSs), they are tested using ASME and ISO standards, with a provision for interim testing through a procedure developed by the National Institute of Standards and Technology (NIST). A central piece of equipment common among these tests is a 2.3 m long scale bar [1, 2].

The scale bar designed for these tests contains at least two kinematic nests that are arranged collinear to each other. Each of these nests is designed to precisely hold and locate a spherically mounted retroreflector (SMR). These bars are usually carefully designed such that they satisfy the design requirements to make them suitable for testing of an LTS [3]. Chief factors among these requirements are that they can be calibrated with an expanded measurement uncertainty (k = 2) to be one fourth of the LTS’s maximum permissible error (MPE), and they must be 2.3 m in length.

A 2.3 m length measurement has long been the minimum required transverse test length to evaluate LTSs, when testing to ASME or ISO standards. However, the NIST-developed interim test showed that a bar with three nests that are 1.15 m apart can still be sufficient to show performance errors of an LTS [4]. In this case, the LTS is placed in such a manner that the 2.3 m length is symmetrical with respect to the LTS, and each of two 1.15 m lengths is asymmetrical with respect to the tracker. However, to achieve a higher test sensitivity, a longer test artifact is necessary. When the user does not have access to a longer artifact, stitching multiple short lengths to achieve a longer one is an acceptable practice. The experiments in this paper will show how the NIST interim test can be modified to utilize a stitched together artifact to achieve a longer test length.

## 2 Overview of NIST Interim Test

**Fig. 1:**
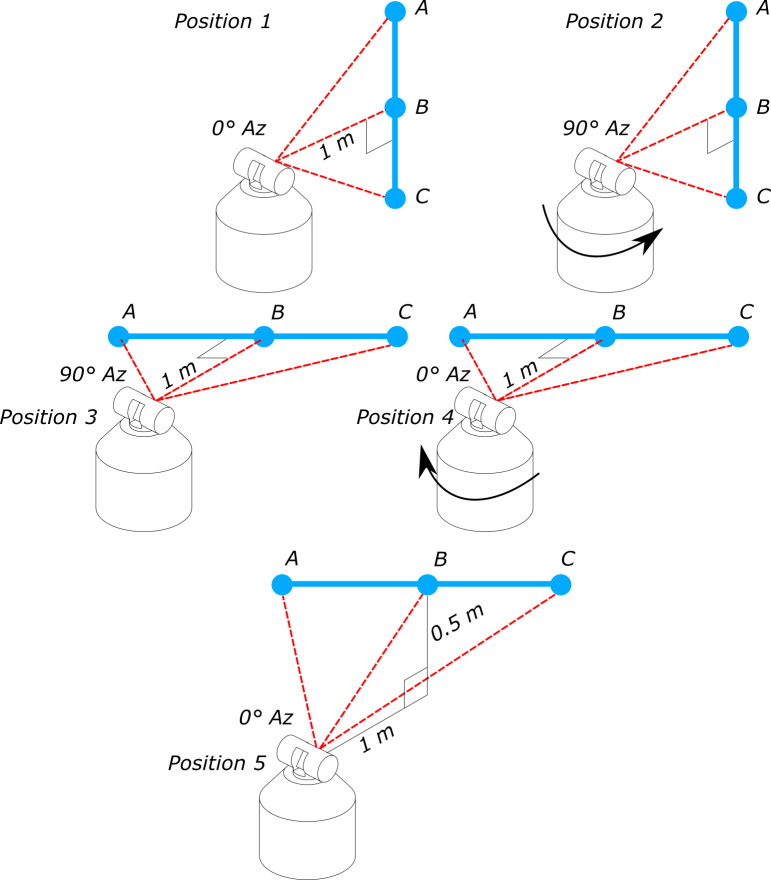
Position of tracker and scale bar for transverse and two-face test measurements [4]. Az = azimuth.

Interim testing of an LTS in accordance with NIST Internal Report (IR) 8016 calls for transverse and two-face measurements of targets at specific locations and positions, as shown in Fig. 1 [4].

Typically, these test locations are embodied through the use of a scale bar with three kinematic nests mounted along its length [3]. An SMR is placed in each kinematic nest located at points "A," "B," and "C." This procedure has been adopted as the recommended interim test in the ongoing revisions of both the ASME B89.4.19 and the ISO 10360-10 standards, but with one small modification. Whereas NIST-IR 8016 required position 2 to also be performed with the scale bar at a distance of about 1 m from the LTS, the user is now free to place the scale bar at any distance so as to capture errors at distances typically encountered in the measurement application for which the LTS is being used.

This interim test is intended to evaluate a sufficient portion of the tracker's measurement volume so that any large errors in the calibration of the device's mechanisms would be revealed as measurement errors of the transverse length and two-face testing. Performing these tests helps to reveal potential calibration errors that cause measurements to exceed the LTS's MPE. This is important so that users do not waste time performing potentially erroneous measurements. To begin, test positions shown in Fig. 1 are realized by calibrating the relative locations of SMRs in locations "A," "B," and "C." Calibration of the scale bar used in the NIST-IR 8016 test is done by aligning the radial measurement component of the LTS such that it is collinear with the nests on the scale bar, also known as a "buck-in measurement." This is done with due care by the user such that the contribution of the angular components of the LTS to the calibration is minimized. These three-nest bars are designed to rotate about location "B" to replicate the test positions cited in NIST-IR 8016. There is one nest located at each end of the scale bar, and one approximately at the center of rotation. Each of these nests is designed to hold an SMR, typically of the 38.1 mm (1.5 inch) variety. These nests are nominally spaced 1.15 m apart to achieve the necessary test positions prescribed in NIST-IR 8016.

Once the scale bar is calibrated, it is oriented in the vertical position, and the tracker is positioned so that its center is at the same height as the central nest, "nest B," and about 1 m away. Next, the locations of nests A, B, and C are measured by the LTS to obtain a two-face error and lengths AB, BC, and AC. Afterward, the tracker is rotated 90° in the counterclockwise direction, when looking down on the tracker, and nests A, B, and C are measured again. This process is done again with the bar in a horizontal position, and finally it is elevated about 0.5 m above the tracker, and the process is done again. Completion of this test typically takes 20 min or less.

## 3 What Interim Testing Can Reveal

One of the intents of the interim test procedure is to quickly verify that an LTS unit is functioning as designed. The results from an interim test are compared to the MPE values claimed by the LTS manufacturer to determine if it is performing within specifications. To demonstrate what typical test results look like, we obtained a commercial off-the-shelf LTS and evaluated it using NIST-IR 8016. For the instrument under test, the stated MPE for two-face error is 90 µm, and the stated MPE for volumetric errors is about 65 µm (newer LTSs will have lower MPEs). After the device was removed from its packaging and allowed to complete its initial warm up cycle, the interim test was performed.

**Fig. 2:**
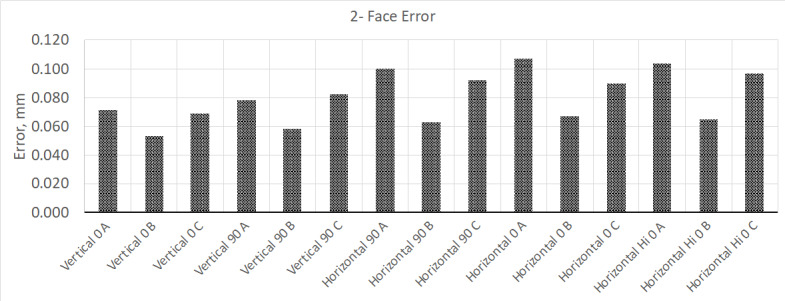
Results from "two-face error" testing, where the MPE for this model LTS is about 0.090 mm, which this device exceeded in several measurement positions.

**Fig. 3:**
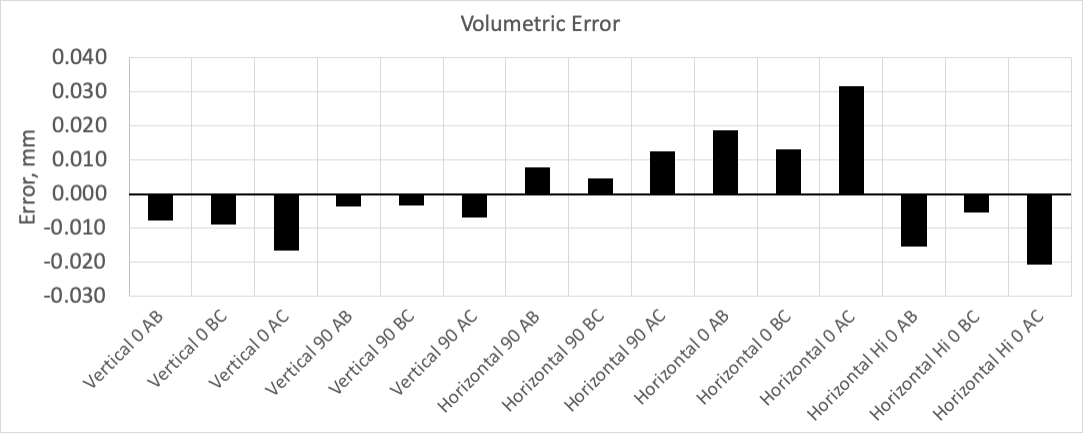
Results from volumetric performance test. This device meets manufacture performance specifications (MPE is ±0.065 mm).

This test was performed using a three-nest scale bar, which was calibrated using the interferometer built into the LTS. Calibration of the scale bar was performed per the manufacturer's recommendation immediately before the test by bucking in the laser tracker so that the measurement laser was collinear with the three nests on the bar. The expanded measurement uncertainty (*k* = 2) from calibration was about 10 µm. The results from this test are shown in Figs. 2 and 3.

**Fig. 4:**
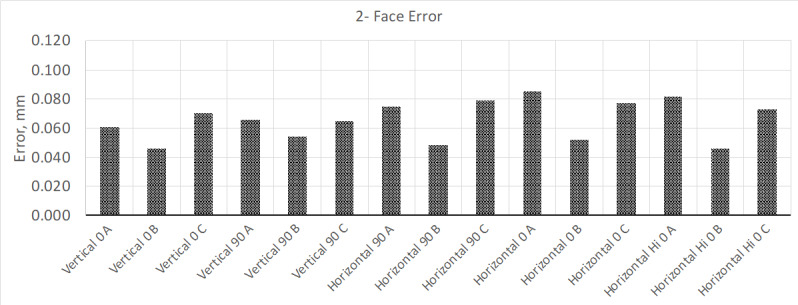
Results from two-face test after allowing the instrument to warm up for 4 h.

From these test results, the two-face tests show that a few of the tests positions exceed MPE. At this point, the manufacturer's compensation routine can be performed to correct for this error. However, the instrument was allowed to warm up for 4 h. Once this time had elapsed, the interim test was performed again. The new results showed that the instrument met the MPE (Fig. 4); volumetric measurement results remained mostly the same.

**Fig. 5:**
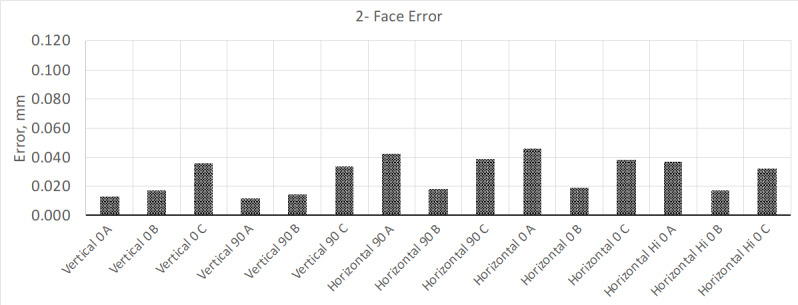
Results from two-face test after allowing the instrument to warm up for 24 h.

Wondering if the results for the two-face test could improve more after a more sustained warmup cycle, the instrument was allowed to warm up for a total of 24 h. Once this extended warm-up cycle was completed, the interim test was performed again, and the results are shown in Fig. 5.

With this additional warm-up time, the instrument's performance on the two-face test improved by a factor of two better than its initial test. Allowing the instrument to warm up for an additional 48 h improved the test results slightly.

One reason why this instrument could display this behavior is because the instrument compensation routine was performed after a substantial warm-up cycle of over 24 h. Since these results were produced from a device that was compensated after a prolonged warm-up period, would compensating the device immediately after the initial warm-up cycle produce similar time-dependent results, but in the reverse direction? That is, would the two-face measurement performance get worse as the device continued to warm up? The following experiment was performed to find out.

**Fig. 6:**
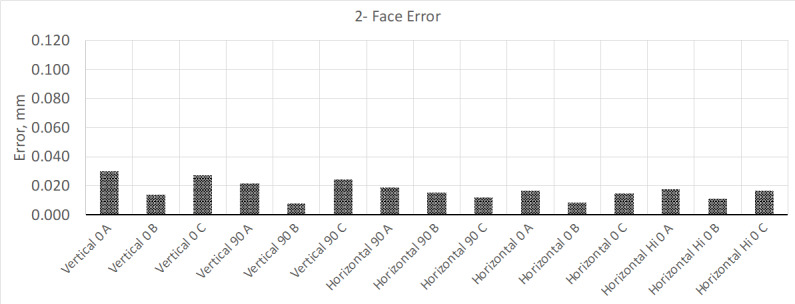
Results from two-face measurement test after initial warm-up cycle and application of compensation routine.

**Fig. 7:**
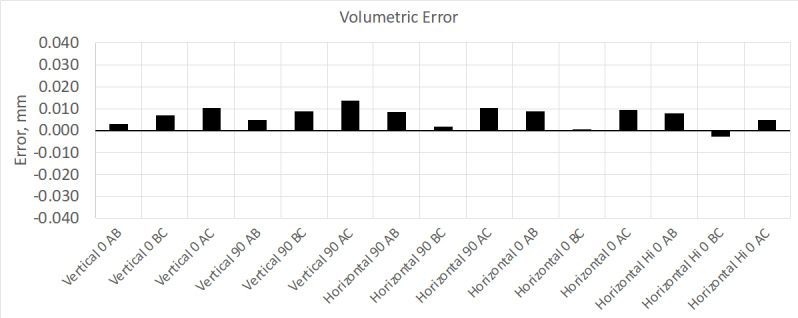
Results from volumetric measurement test after initial warm-up cycle and application of compensation routine (MPE is ±0.065 mm).

To begin, the same instrument used in the earlier test was shut down and allowed to cool for 24 h. Subsequently, it was turned on and allowed to complete its initial warm-up cycle. No other changes were made to the setup, the laboratory temperature was kept at 20 °C ± 0.5 °C, and the position of all the equipment remained the same. Next, the manufacturer's compensation routine was completed, the interim test was performed, and the results are shown in Figs. 6 and 7.

**Fig. 8:**
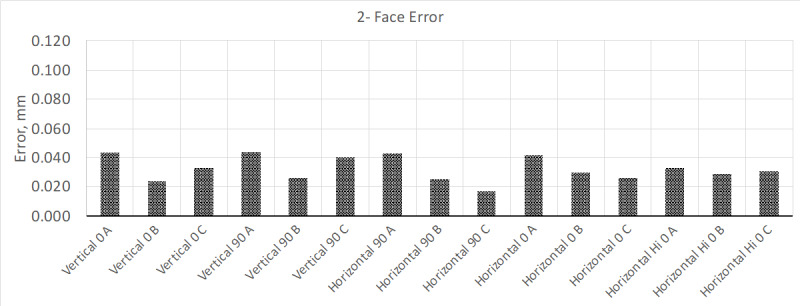
Results from two-face measurement test after 4 h of warm up.

After initial warm up, compensation of the tracker, and running the interim test, all of the test results showed that the device was performing well within MPE specifications, as expected. Next, the instrument was allowed to warm up for four additional hours and retested, with the results for the two-face test shown in Fig. 8. The results for the volumetric portion of the test did not show any significant change.

**Fig. 9:**
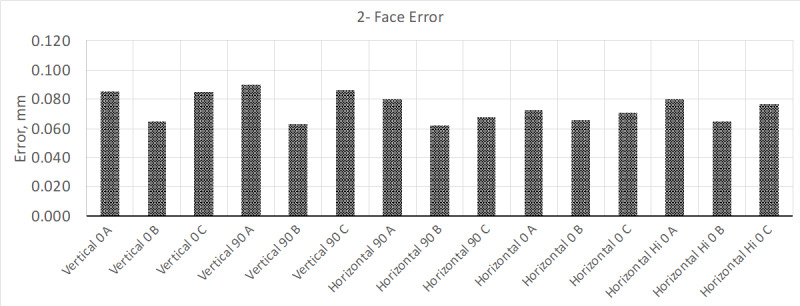
Changes in two-face test performance after warming device up for 24 h.

**Fig. 10:**
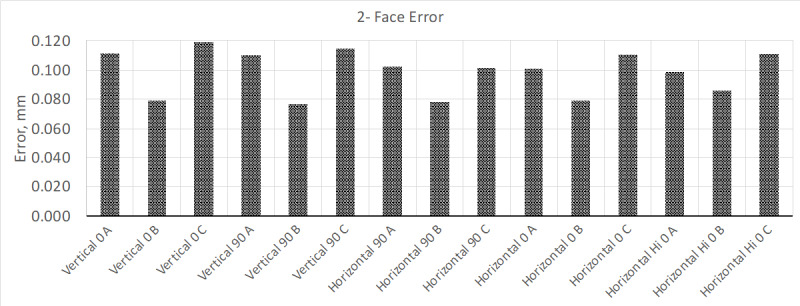
Changes in two-face test performance after warming device up for 48 h.

**Fig. 11:**
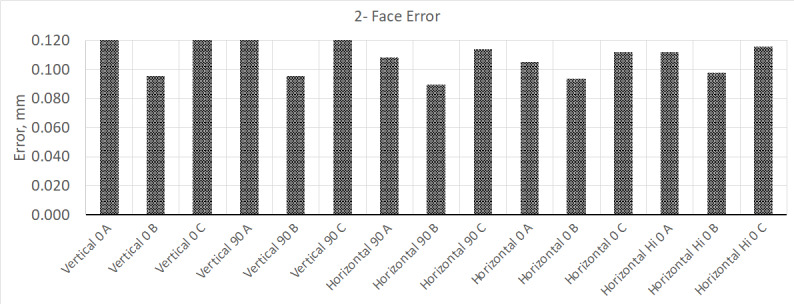
Changes in two-face test performance after warming device up for 72 h.

Unlike the previous test, where the measurement results improved after 4 h of warm up, this time the magnitude of the two-face test errors increased but remained under the MPE. Allowing the instrument to warm up further for a total of 24, 48, and finally 72 h yielded the following results (Figs. 9, 10, and 11, respectively).

As the device progressively warmed up, the two-face test results continued to increase, until they exceeded the MPE specifications for this instrument; this occurred sometime between 24 and 48 h of run time. Knowing that this instrument behaves in this manner, the user can devise a schedule on how often to perform the instrument compensation routine, until it reaches a steady-state operating condition. For example, it may be advisable to run the compensation routine every 4 h until it reaches steady-state operation. From all these tests, it is conceivable that an instrument can start out producing measurement results that are within MPE specifications but exceed it as it is used throughout the day(s), or vice versa.

The testing described in the preceding sections shows how a quick interim test can provide information to inform the user on the transient behavior of their instrument. However, if users wanted to stitch together measurement results to achieve the necessary test lengths, how could they do that, and what would those results look like?

## 4 Stitching Scale Bar Method

**Fig. 12:**
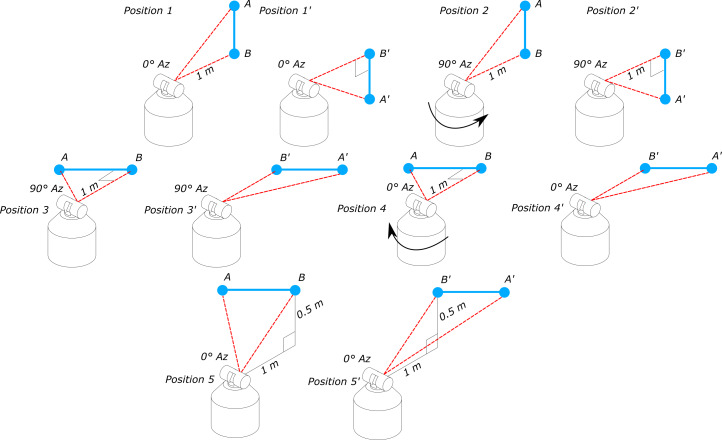
Modification of test positions using shorter scale bar.

When performing interim tests, users may elect to use longer test lengths to evaluate their device. On other devices, such as a terrestrial laser scanner, a test length as long as 6 m or more may be needed [5, 6, 7]. Individual test lengths that far exceed 2 m in length can be unwieldy, cumbersome to use, and challenging to calibrate. For users that need to realize a test length that is longer than any scale bar they currently have on hand, stitching measurement results of shorter artifacts to span the necessary length could be a viable option. To evaluate this concept, NIST-IR 8016 can be modified to use a shorter length that can be stitched together to achieve long test length equivalents. For example, to evaluate the LTS measurement zone for position 1 in Fig. 1, the short scale bar starts out spanning the distance between targets "A" and "B" and then is shifted to span the measurement zone between targets "B" and "C." Instead, targets A' and B' would replace B and C from the normal method. This would be repeated for all the test positions until the required test positions and two-face tests are replicated (Fig. 12).

With the revision of the interim test using the stitching method, the test positions and the required number of tests remain unchanged; the added bar manipulation does add a few minutes to the test. Realizing an equivalent test result for the longer (symmetrical) test length (length AC of the original test in Fig. 1) will require summing up the results from lengths AB and A'B' from each position; see Fig. 12. Thus, the error in the length AC in Fig. 1 can be realized as the sum of the errors of the two segments AB and B'A' in Fig. 12. Care needs to be taken so that points B and B' are coincident to within 2 mm, so that the measurement zone being evaluated closely matches that of the original NIST-IR 8016 symmetric test length AC.

**Fig. 13:**
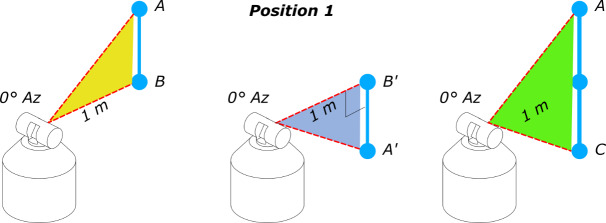
Swept distance from targets A to B plus B' to A' covers the same swept distance as measuring from target A to C in the original long test length setup.

Researchers have shown that LTS errors slowly vary across the measurement volume, and so the point coordinate errors of points B and B? are almost identical, even if they are, say, a few millimeters apart from each other [7, 8]. In other words, the results from the two independent measurements of the short bar (AB and B'A') could be summed to derive an equivalent result for a hypothetical long test length (AC) as shown in Fig. 13.

An additional requirement to ensure that the sum of the individual errors is equal to the error of measuring an equivalent long length bar is that the two lengths need to be nearly collinear with each other with a straightness of less than 5 mm.

**Fig. 14:**
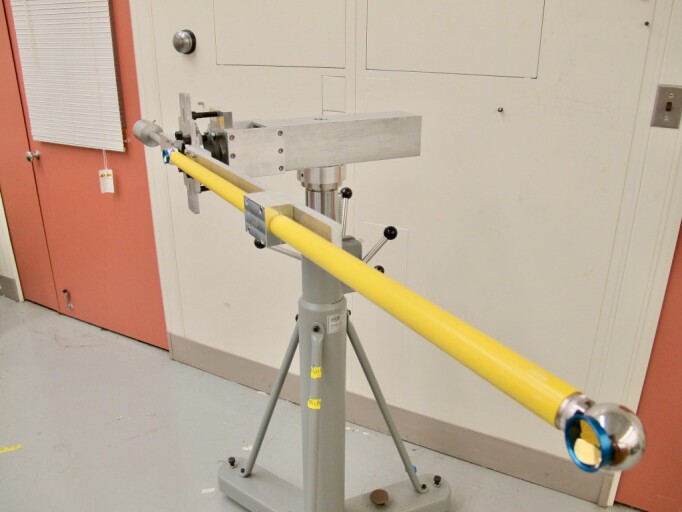
Test fixture for 1.0 m scale bar.

**Fig. 15:**
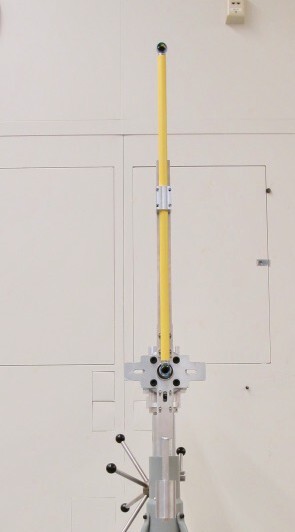

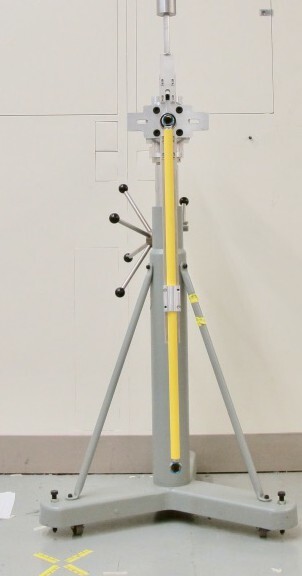

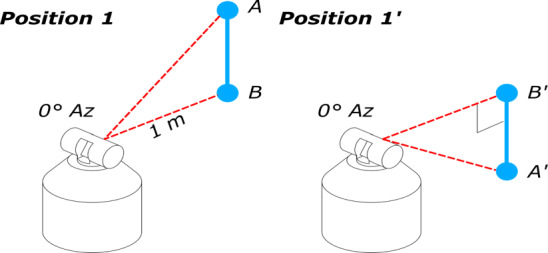
Manipulation of the short bar to span the long test length. SMRs are attached to the ends of the scale bar.

To embody a design concept that utilizes this revised test, a rotary mechanism was constructed that allows the user to index a short scale bar into the positions shown in Fig. 12. The design nominally places one SMR (target B/B' in Fig. 12) of the scale bar about the axis of rotation of the mechanism, while the other end can rotate around the axis. A commercial off-the-shelf automotive wheel bearing provides the mechanism of rotation, and threaded holes are used to mount the fixture that holds the scale bar. The fixture was designed to hold a two-nest Invar^1^1Certain commercial equipment, instruments, or materials are identified in this paper in order to specify the experimental procedure adequately. Such identification does not imply recommendation or endorsement by the National Institute of Standards and Technology, nor does it imply that the materials or equipment identified are necessarily the best available for the purpose. scale bar that is nominally 1 m in length, and each nest holds a 38.1 mm (1.5 inch) diameter SMR. The design allows the scale bar to be indexed in the vertical and horizontal positions needed for the interim tests, and in positions that allow the stitched lengths to be collinear with each other such that they have a straightness of 5 mm (Figs. 14 and 15). Target B/B' is positioned near the rotary axis such that it does not move more than 2 mm when the bar is rotated.

Although 1.15 m and 2.3 m test lengths are recommended in the NIST-IR 8016 procedure, one could not be obtained in time for these tests. In its place, two measurements of a 1.0 m test length were then stitched together to derive a 2.0 m test length, which was used for proof of concept.

## 5 Testing the Stitched Scale Bar Concept

**Fig. 16:**
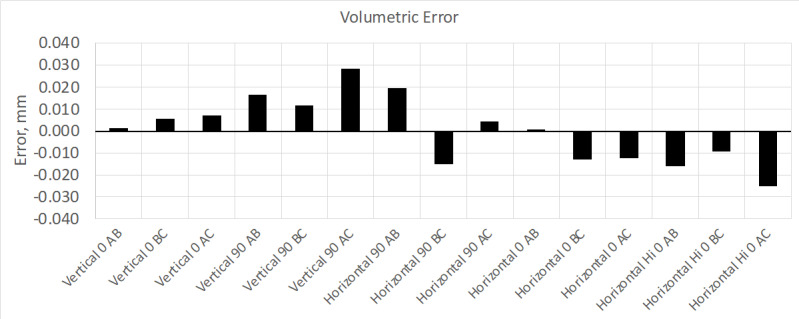
Volumetric error using the stitched scale bar method (MPE is ±0.065 mm).

**Fig. 17:**
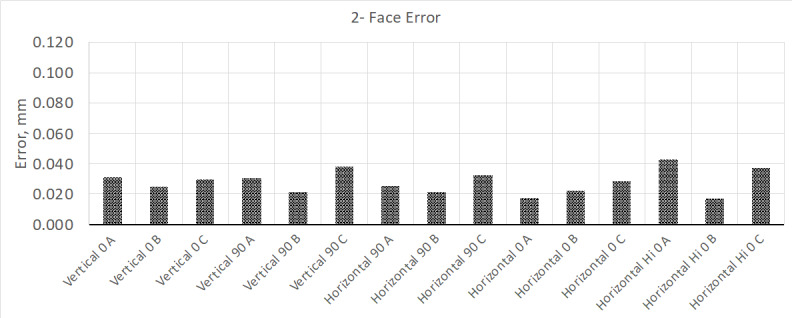
Two-face error results from measurements using the stitched scale bar method (MPE is ±0.090 mm).

The prototype shown in Figs. 14 and 15 was used to understand if the NIST interim test could be performed using a short scale bar stitched together to approximate a long scale bar. To capture the necessary data to perform the tests prescribed in NIST-IR 8016, the modified test procedure shown in Fig. 12 was used. In the normal interim test case, measurement results of the short and long test lengths can be directly compared to their calibrated values. In the case with the stitched together scale bar, only one calibrated length is available to stand in for the short test lengths. The long test lengths would be realized by stitching the measurement results together from the two independent measurements in the proper orientations; for an example, please see Fig. 13. The following figures show typical test results for measuring a short scale bar using the modified test methods (stitching together two short lengths to obtain an equivalent AC length; see Figs. 16 and 17). These measurement errors are comparable to those obtained using the normal interim test method shown in the previous test results (Figs. 2 and 3).

**Fig. 18:**
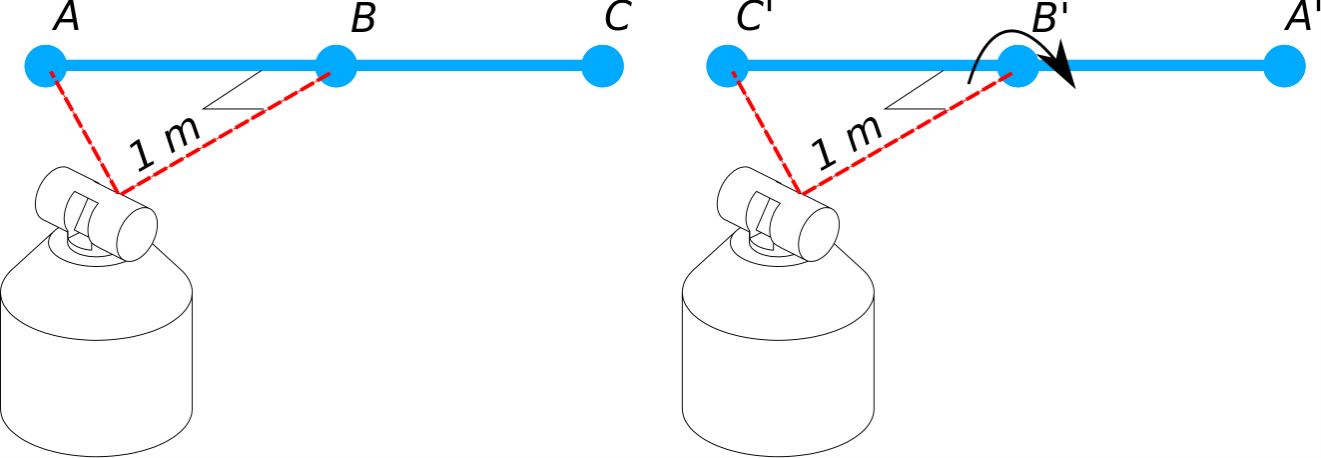
Evaluating the same measurement zone, but with a slightly different length.

To verify that the stitching and standard methods (*i.e*., using a short single bar) produce repeatable results, a three-nest scale bar was measured in a manner that evaluates this simultaneously. When the LTS measures the scale bar, it is evaluating a particular measurement zone. If the LTS is performing as designed, its length measurement error should be within its MPE, and it should be repeatable for each zone. With the three-nest scale bar, lengths AB and BC differ by 65 µm, and the imperfect motion of the rotary bearing causes about 2 mm of runout when indexed to 180°. Taking advantage of this, each measurement zone of the interim test for a given test position can be evaluated by a slightly different length, and at a slightly different position. For example, in position 3 of the interim test, the measurement zone that is evaluated by test length AB can be evaluated again by length B'C' (which differs by 65 µm) by rotating the three-nest scale bar by 180° (Fig. 18).

**Fig. 19:**
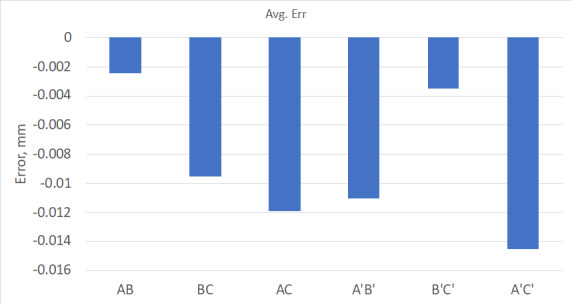
Average error from modified interim test.

The same can be said for the measurement zone evaluated by test lengths BC and AC. So, if the short stitched scale bar concept functions as expected, and the performance of the LTS at each measurement zone is repeatable and slowly varying as mentioned earlier, the measurement results (*i.e*., length errors) will show the following:

(1) LTS errors from measuring lengths AB and C'B' will nominally be the same.
(2) LTS error from measuring lengths BC and B'A' will nominally be the same.
(3) LTS errors from measuring lengths AC and C'A' will nominally be the same. This test was performed six times, and averaged results are shown in Fig. 19.

As shown in Fig. 19, this LTS measuring slightly different lengths in the same measurement zone produced similar results (*e.g*., length error AB is roughly the same as B'C'). By measuring the three-nest scale bar in this manner, the tracker is exposed to three independently calibrated lengths in a different orientation, similar to flipping the short scale bar 180° to complete a stitched test length evaluation. Despite the 65 µm length differences and 2 mm shift, when these lengths are presented to the tracker to evaluate the same portion of the tracker's measurement zone, the results are nominally the same. Since the laser tracker is repeatable, and its errors are slowly varying, slight differences in test length and position did not make a significant difference in its measurement performance; 2 µm to 3 µm of difference is within the nominal repeatability of the laser tracker's performance.

## 6 Measurement Uncertainty of a Short Scale Bar

Following the 4:1 simple acceptance decision rule, for a test length to be suitable for interim testing, its expanded measurement uncertainty needs to be one quarter of the device's MPE, in this case, 16 µm (65/4 µm) [9, 10]. For the short scale bar used in the preceding experiments, it was calibrated using a Cartesian coordinate measuring machine (CMM). Table 1 outlines its calibration uncertainty and sources, with each one described as follows.

## 6.1 SMR Mounting Repeatability

With the kinematic nests used on this scale bar, the typical variation on the absolute position of the SMRs from repeated mounting and dismounting ranges ±1 µm. Taking this value as the bounds of the expected length variation due to this source of uncertainty, and assigning this value a rectangular probability distribution, its expected contribution to standard uncertainty is about 0.6 µm.

## 6.2 SMR Centering

The apex of the SMR's corner cube will not perfectly coincide with the geometric center of the sphere in which it is mounted. Since the CMM measures the distance between the geometric centers of the outer body of the SMRs mounted on the ends and not the corner cube locations, this misalignment between centers will have an effect on the calibrated value. As supplied by the vendor, this misalignment is about ±2.5 µm. If this value is treated as the extreme bounds of the expected misalignment, and a rectangular probability distribution is assigned to this bound, it is anticipated that this will contribute about 1.4 µm to the standard measurement uncertainty of the scale bar.

## 6.3 SMR Form Variation

The spheres used to construct SMRs can have a form that varies by as much as ±1.27 µm, consistent with a grade 50 ball. This form variation is especially pronounced near the opening of the SMR. When the nest impinges on the sphere surfaces near its opening, it can cause a shift of about ±2.3 µm. If this value represents the expected range in bar length difference due to SMR diameter variation, and a rectangular probability distribution is assigned to this range, then this value's expected contribution to standard measurement uncertainty is about 1.3 µm.

## 6.4 CMM Length Measurement

This scale bar was calibrated on a CMM by measuring 25 points on the spherical side of the SMR. From these points, a least squares sphere fit was performed to find their geometric center, and the length of the bar was found by calculating the distance between these two centers. From 12 repeated measurements of the bar, a standard deviation for the calibrated length of 0.45 µm was calculated. To compensate for any systematic error for unidirectional length measurements, a 1 m long step gauge calibrated with a standard uncertainty of 0.155 µm was measured. Summing these two values in quadrature yielded a standard uncertainty of about 0.5 µm.

## 6.5 Bar Bending Effects

Since the SMRs are not perfectly aligned with the neutral bending plane of the scale bar, its orientation to gravity will have an effect on its effective length [11]. To account for this, the scale bar was calibrated in two orientations using the bar's serial number as a reference for orientation. In one orientation, it was calibrated with the number facing up, and in another orientation, it was calibrated with the number facing down. These values were averaged to obtain a value for the scale bar's length. Between these two orientations, the length of the bar varied by a range of about ±0.75 µm. If this value represents the expected range in bar length variation due to bending effects, and a rectangular probability distribution is assigned to this range, then this value's expected contribution to standard measurement uncertainty is about 0.4 µm.

## 6.6 Temperature Variation

When this scale bar is placed in the laboratory, its temperature will vary by ±0.5 °C from a nominal set point of 20 °C. The scale bar is made from Invar, which has a nominal coefficient of thermal expansion of 1.3 µm/(m °C). If the bar is expected to experience a range in temperature change that does not exceed what is noted above, and a rectangular probability distribution is assigned to this range, then the expected standard uncertainty due to temperature variation is about 0.4 µm.

**Table 1:** Calibration uncertainty of a short scale bar.

Uncertainty Source	Value (µm)
SMR Mounting Repeatability	0.58
SMR Centering	1.44
SMR Diameter Variation	1.30
CMM Length Measurement	0.54
Bar Bending Effects	0.43
Temperature Variation	0.38
Standard Measurement Uncertainty	2.17
Expanded Measurement Uncertainty (*k =* 2)	4.35

Taking all of these uncertainty sources and summing them in quadrature, the expanded measurement uncertainty (*k =* 2) is 4.35 µm for a single span of the short scale bar. When the short scale bar is stitched to achieve longer test lengths, this uncertainty value is multiplied by the number of times the bar is stitched to calculate the measurement test uncertainty. In the case presented here, the scale bar was stitched twice to produce the 2 m long test length, so the expanded measurement uncertainty for that test length would be approximately 8.7 µm.

## 7 Concluding Remarks and Future Work

Utilizing a short test length to realize longer test lengths via stitching has long been an acceptable practice to evaluate CMM performance. This paper has shown that this method can also be applied to portable CMMs such as a laser trackers. This capability can help users that do not have the space or budget for calibrated lengths that far exceed 2 m in length. Shorter artifacts can also take advantage of off-site calibration methods such as using a CMM. Future work will show how stitching short scale bars can be applied to terrestrial laser scanners and how this method compares to traditional methods.
